# Incidence of Rhegmatogenous Retinal Detachment (RRD) in a Tertiary Care Center of Pakistan

**DOI:** 10.7759/cureus.25092

**Published:** 2022-05-17

**Authors:** Saad M Iqbal, Kashif Iqbal, Arslan Shahid, Faisal Iqbal, Fawad U Rahman, Mohammad J Tahir, Zaheeruddin A Qazi, Usama Raheem, Jawad B Butt, Moiz Ahmed

**Affiliations:** 1 Ophthalmology, Layton Rehmatulla Benevolent Trust (LRBT) Free Eye Hospital, Lahore, PAK; 2 Ophthalmology, Sargodha Medical College, Sargodha, PAK; 3 Ophthalmology, D.G. Khan Medical College, Dera Ghazi Khan, PAK; 4 Ophthalmology, Layton Rehmatulla Benevolent Trust (LRBT) Free Eye Hospital, Karachi, PAK; 5 Cardiology, National Institute of Cardiovascular Diseases, Karachi, PAK; 6 Medicine and Surgery, Sindh Medical College, Karachi, PAK

**Keywords:** rhegmatogenous, retina, optical, ocular trauma diabetes mellitus, detachment

## Abstract

Background

Regardless of the advancements in ophthalmology, rhegmatogenous retinal detachment (RRD) remains a substantial issue for physicians. The present study assessed the incidence of RRD among our population.

Methodology

A cross-sectional study was performed at the Layton Rehmatullah Benevolent Trust (LRBT) between June 2020 and May 2021. All the patients of RRD, irrespective of gender, within the age bracket of 20 years or more and diagnosed by a consultant ophthalmologist were included in the research study. Patients with serous retinal or tractional detachment and RRD with vitreous leakage were excluded from the study. A slit lamp and dilated fundus examination was performed preoperatively to assess the type of retinal detachment and associated factors as mentioned above. All data were collected on predesigned pro forma.

Results

About 25,000 individuals were presented to the outpatient department during the study period. Out of these, 100 patients were diagnosed with RRD. The incidence rate of the RRD in our center was 0.4%. There were a majority of the males. The mean age of patients did not vary significantly with respect to gender (p < 0.797). The most common type of RD was the total RD with a frequency of 53 cases followed by inferior RD with 19 cases. The majority of those with total RRD were males, i.e., 37%; however, the difference was statistically insignificant (p = 0.476). The study revealed that most of the RRD was diagnosed in patients < 45 years of age; however, the difference was not statistically significant (p < 0.227).

Conclusion

The present study highlighted the incidence of RRD and explored the sociodemographic and other clinical features in the Pakistani population. However, it is possible that the RRD condition is still under-diagnosed in our hospital settings. Further exploration is warranted to study comprehensively the risk factors associated with RRD.

## Introduction

Rhegmatogenous retinal detachment (RRD) is one of the most common types of retinal detachment (RD), which occurs due to a retinal tear, which results in the separation of the retinal pigment epithelium and neurosensory retina. However, regardless of the advancements in ocular treatments, achieving functional recovery is limited and only 42% of patients regain a 20/40 vision, while only 28% of patients with a damaged macula restore vision [1-3].

Even minor mechanical pressure such as those elicited by excessive intraocular fluid or interdigitations between microvilli and photoreceptors may cause the two layers to have adhered together [4]. RDs subsequently result from alterations in these adhesive forces. In addition to RRDs, other types of RDs include exudative, tractional, and mixed tractional/RRDs, each of which has a distinct pathology. RRD most commonly occurs secondary to atrophic perforation or simple tears in the region of vitreoretinal attachments during the break of the posterior vitreous detachment (PVD) [5].

Individuals with RRD frequently experience flashes of light or eye floaters, which are gradually proceeded by a loss of visual field [6]. These individuals might have pigmented cells in the vitreous region. The most frequent location is the anterior chamber. The underlying detached retina is non-transparent with a ridged surface and often changes its direction with the movement of the eye. Retinal breaks usually exist, even if they are not necessarily observable. The retinal break must be closed surgically to be treated. If neglected, symptomatic RRD will always culminate in visual loss [7].

Literature shows a high occurrence of RRD among the elderly population, while other studies also indicate a bimodal distribution and a subsequent peak at younger ages due to significant myopia [8]. RRD was reported to have the greatest incidence rate among people aged 60 to 69 years, with rates ranging from 19 to 27 per 100,000 individuals [9]. Due to the scarcity of local literature, the present study was undertaken in our setting. The goal of the study was to evaluate the frequency of RRD among our population.

## Materials and methods

A cross-sectional study was performed at the Layton Rehmatullah Benevolent Trust between June 2020 and May 2021. Before starting data collection, the study was approved by the ethical committee with reference # GEN-LRBT/866. The participants were recruited using the non-probability convenience sampling method. 

All patients who presented to the ophthalmologist at our center were included in the research. Patients were examined thoroughly by a senior consultant. Those who were positively diagnosed by an ophthalmologist for RRD were recruited for the final analysis. Patients of RRD with vitreous leakage and RD were also eliminated. Before initiation of data collection, patients were informed and verbal consent was obtained. The goals of the study were clearly narrated to all the patients. This step was followed by the acquisition of demographic data relevant to age, gender, and ethnicity for adequate documentation. The visual acuity was also documented using the Snellen chart. The patient was requested to occlude the non-affected eye and start reading the chart as far as possible. Visual acuity was documented for the affected eye. Using the classification of visual disability by the World Health Organization, the patients were categorized as having mild (below 6/12 to 6/18), moderate (below 6/18 to 6/60), severe visual acuity (< 6/60 to 3/60), and blindness (< 3/60) [10].

Patients’ attendants were interviewed to obtain prior history while a diagnostic report was obtained to verify cataract surgery. A slit lamp and dilated fundus examination were conducted to assess the nature of RD and related variables, as stated earlier. All of the information was gathered on already prepared questionnaires. A total of 25,000 patients were presented to the outpatient department of ophthalmology during the study period. Out of these 100 patients were diagnosed with RRD.

For statistical analysis, the statistical package for social sciences and Microsoft excel were employed to determine the descriptives for the variables. Mean and standard deviation was quantified for continuous variables. For ordinal and nominal characteristics such as gender and eye involvement, frequency and percentages were assessed. The association of total RD was assessed with the demographic and clinical parameters of the patients using the chi-square test. A p-value of below 0.05 was decided as the cut-off value for statistical significance. All data were presented as tables and figures. Images of some cases were also provided.

## Results

The incidence rate of the RRD in our center was 0.4%. The most common cause of RRD was lattice degeneration which was observed in 36 cases while ocular trauma was established as the cause of RRD in 23 cases. Furthermore, in the majority of the patients, the right eye was involved, and the majority were younger than 45 years old. The majority of the patients had severe visual impairment highlighting visual acuity worse than 6/60 to 3/60 (Table 1).

**Table 1:** Demographic and clinical characteristics of the study participants RD: retinal detachment

Characteristics	Frequency (%)
Age Group	
<45 years	57
45 years and above	43
Gender	
Male	68
Female	32
Eye involved	
Right	60
Left	39
Impairment of Vision (Visual Acuity)	
Mild impairment	1
Moderate impairment	4
Severe impairment	76
Break Location	
Inferior + Temporal Breaks	1
Inferior + Temporal Lattice With Holes	1
Inferior Break	5
Inferior Horseshoe Break	7
Inferior Lattice with Holes	14
Inferotemporal Horseshoe Break	4
Inferonasal Break	3
Infratemporal Break	1
Infratemporal Break	1
No Break Found	3
Superior Break	14
Superior Horseshoe Break	14
Superior Lattice with Holes	6
Superotemporal Break	18
Temporal Break	8
Causes of retinal detachment	
Complicated phaco	11
Extracapsular cataract extraction	2
Iatrogenic	1
Intracapsular cataract extraction	1
Lattice degeneration	36
Post intravitreal injection	1
Posterior vitreous detachment	1

**Figure 1:**
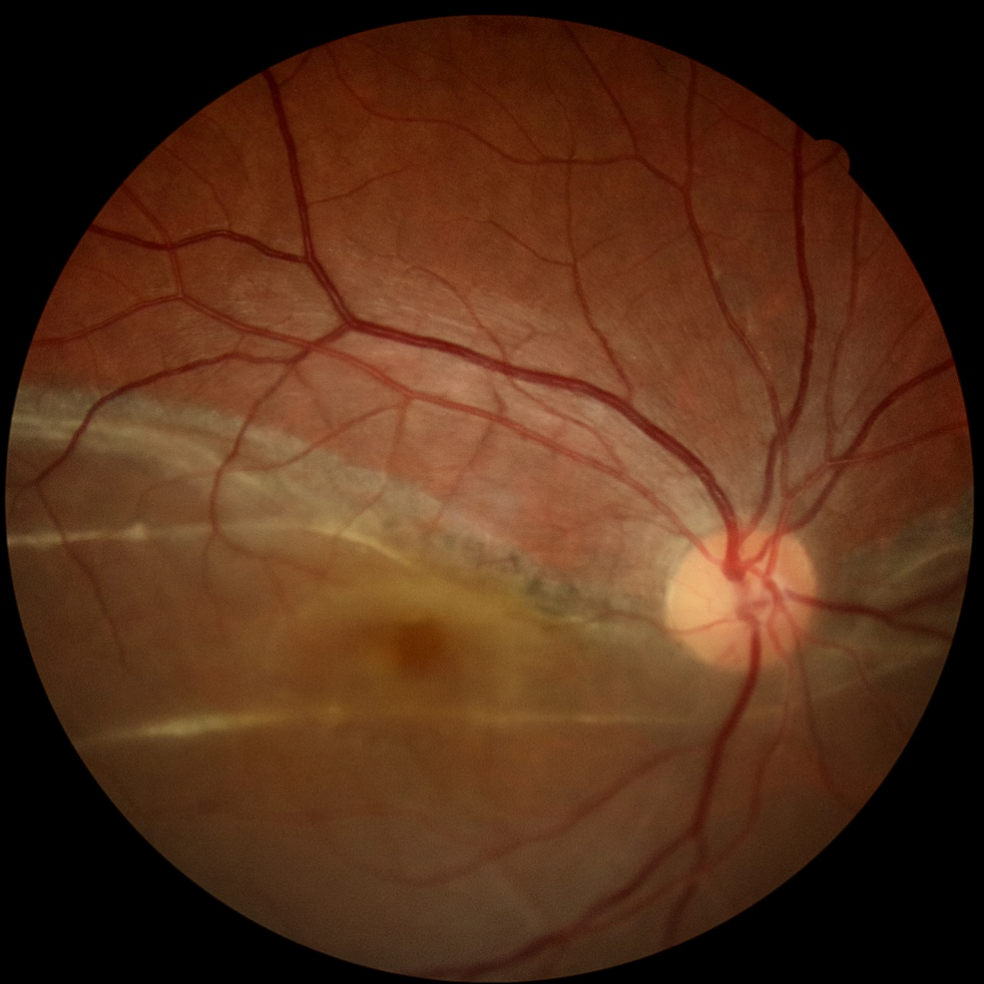
Inferior retinal detachment in the right eye

**Figure 2:**
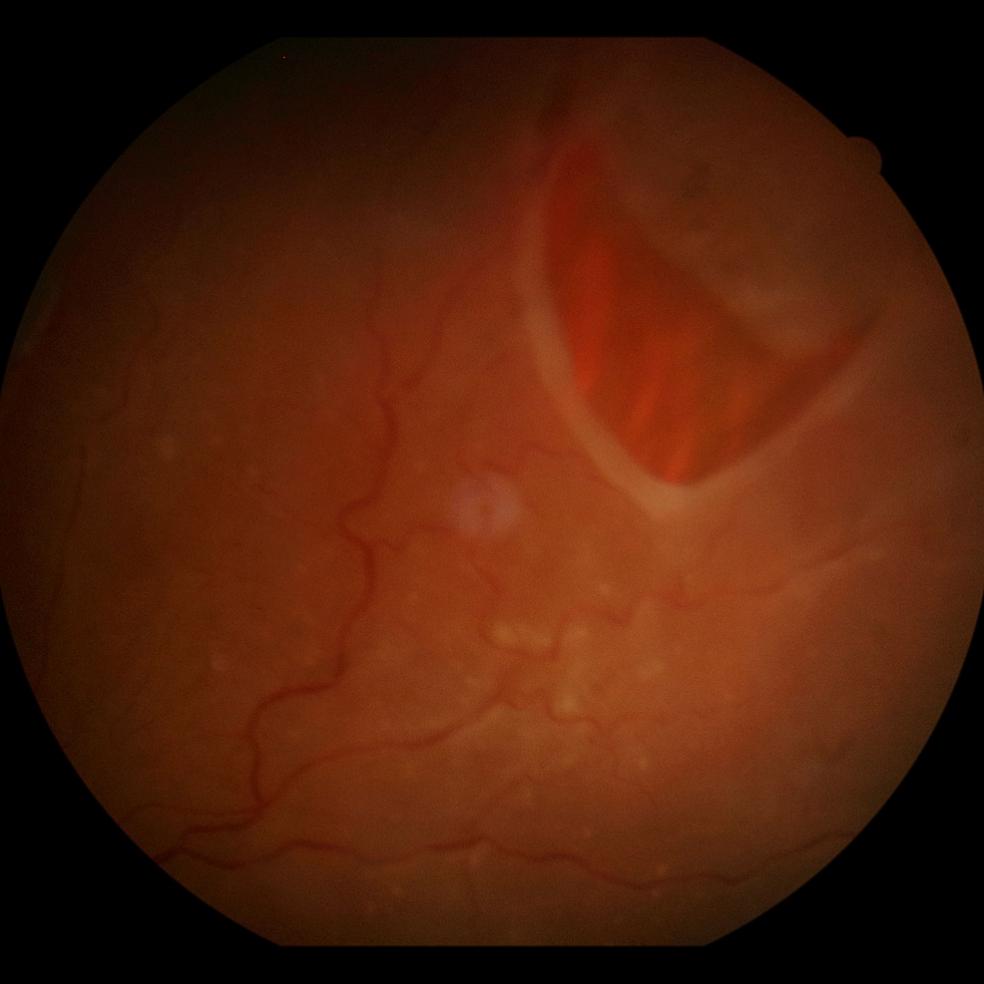
Total retinal detachment in the right eye

There was a predominance of male patients. The mean age of patients was 37.84 ± 18.29 years. The most common type of RD was the total RD with a frequency of 53 cases followed by inferior RD with 19 cases (Figure 1). Figure 2 illustrates a case with a superonasal horseshoe-shaped break in total RD. 

Further stratification with respect to the total RD revealed no significant impact of age, gender, location of the break, and causes of the RD (Table 2).

**Table 2:** Association between total retinal detachment with demographic and clinical parameters RD - retinal detachment

Characteristics	Yes	No	P-value
Age Group			
<45 years	32	25	0.687
45 years and above	22	21	
Gender			
Male	17	15	0.537
Female	37	31	
Eye involved			
Right	24	15	0.293
Left	30	31	
Break Location			
Inferior + Temporal Breaks	1	0	0.375
Inferior + Temporal Lattice With Holes	1	0	
Inferior Break	2	3	
Inferior Horseshoe Break	2	5	
Inferior Lattice with Holes	6	8	
Inferotemporal Horseshoe Break	1	3	
Inferonasal Break	2	1	
Infratemporal Break	0	1	
Infratemporal Breaks	1	0	
No Break Found	2	1	
Superior Break	8	6	
Superior Horseshoe Break	9	5	
Superior Lattice with Holes	6	0	
Superotemporal Break	10	8	
Temporal Break	3	5	
Causes of RD			
Complicated phaco	7	4	0.486
Extracapsular cataract extraction	1	0	
Iatrogenic	1	0	
Intracapsular cataract extraction	0	1	
Lattice degeneration	17	19	
Post intravitreal injection	1	0	
Posterior vitreous detachment	1	0	
Refractive surgery	0	2	
Ocular trauma	14	9	
Uncomplicated phaco	9	8	
YAG Capsulotomy	1	3	

## Discussion

RD is an ocular condition associated with blindness, which requires prompt treatment to restore function [11]. Our study concluded that the total incidence of RRD was 0.4%. RRD was reported to be the most commonly occurring type of RD by Ohman et al. who reported one in 10,000 cases each year [12]. However, the incidence in our study population is much higher than a similar study by Qureshi et al. who found the incidence of RRD between 0.01% and 0.02% [13]. This discrepancy may be due to variability in age groups in the study population. A higher risk of RDt is associated with an increase in age and is attributed to age-related pathologies predisposing to this condition.

Our study reported only one case of PVD, which has been reported to be a common cause of RRDs. In contrast to our finding, a survey by Seider et al. evaluated the association between a PVD and RRD involving 8,305 patients suffering from PVD. It was found that 4.0% of the patients also suffered from RRD in addition to PVD, thereby concluding that PVD is a risk factor associated with RRD, which is a finding inconsistent with our study [14]. Multiple other reports have been published, according to which the incidence of RRD associated with PVD was found to be between 15% and 27.1% [15].

According to our study, cases of RRD were most commonly seen in the male population. A male predominance is also observed in the studies on RD by Li et al. and Nielsen et al. [16,17]. The difference in gender could be attributed to the anatomical difference in the male and female eyes. According to Olsen et al., male predominance is associated with the male eyes being much longer than females, which is a significant risk factor for RRD [18]. In a thorough systematic review, an epidemiological analysis of RRD was performed by Mitry et al. [19]. It was revealed that RRD is correlated with a substantial geographical variation. Furthermore, the incidence rate was observed to vary between 6.3 and 17.9/100,000 population. The prevalence of lattice degeneration was reported to be approximately 45% [19]. In another recent comprehensive cohort study, the impact of the COVID-19 pandemic was assessed on the burden and clinical course of RRD. It was found that the patients who presented during the pandemic reported worse median visual acuity than prior to the pandemic (p=0.008) [20]. This is in line with our findings where the majority that is approximately 76% patients presented with severe visual impairment (Table 1). 

Our study reported that prior cataract surgery was linked with a greater risk of developing RRD. According to Storey et al., a large chunk of RRD cases are seen in patients with a positive history of cataract surgery [21], with cases ranging from 21.6% to 37.2% [22,23] of RDs. Thus, since males are inherently more susceptible to RRD, male patients who have also undergone cataract surgery have a greater absolute risk of RRD than women [24]. A study by Day et al. elaborated that RRD may occur secondary to cataract surgery, especially in cases where the eye had suffered intraoperative trauma, such as posterior capsule rupture, or if the surgeon was less experienced [25].

Our study was limited due to the small sample size. The study findings cannot be generalized to a larger population. Further studies are required to explore the epidemiology of RRD in a larger population so that practical conclusions can be drawn.

## Conclusions

The current study explored the incidence rate of RRD and found out that the study reported a fairly low incidence rate. However, it is suspected that the current study may have under-reported the prevalence of RRD. Therefore, further multi-center research is required to ascertain the actual epidemiological picture of the Pakistani population.
